# To add or not to add a new treatment arm to a multiarm study: A decision‐theoretic framework

**DOI:** 10.1002/sim.8194

**Published:** 2019-05-21

**Authors:** Kim May Lee, James Wason, Nigel Stallard

**Affiliations:** ^1^ MRC Biostatistics Unit University of Cambridge Cambridge UK; ^2^ Institute of Health and Society Newcastle University Newcastle upon Tyne UK; ^3^ WMS ‐ Statistics and Epidemiology University of Warwick Coventry UK

**Keywords:** adding‐arm, disjunctive power, multiarm, number of rejected hypotheses

## Abstract

Multiarm clinical trials, which compare several experimental treatments against control, are frequently recommended due to their efficiency gain. In practise, all potential treatments may not be ready to be tested in a phase II/III trial at the same time. It has become appealing to allow new treatment arms to be added into on‐going clinical trials using a “platform” trial approach. To the best of our knowledge, many aspects of when to add arms to an existing trial have not been explored in the literature. Most works on adding arm(s) assume that a new arm is opened whenever a new treatment becomes available. This strategy may prolong the overall duration of a study or cause reduction in marginal power for each hypothesis if the adaptation is not well accommodated. Within a two‐stage trial setting, we propose a decision‐theoretic framework to investigate when to add or not to add a new treatment arm based on the observed stage one treatment responses. To account for different prospect of multiarm studies, we define utility in two different ways; one for a trial that aims to maximise the number of rejected hypotheses; the other for a trial that would declare a success when at least one hypothesis is rejected from the study. Our framework shows that it is not always optimal to add a new treatment arm to an existing trial. We illustrate a case study by considering a completed trial on knee osteoarthritis.

## 1 INTRODUCTION

In recent years, a multiarm multistage design is becoming an attractive alternative to the classical randomised treatment‐control design, especially in phase II and phase III trial settings. A multiarm multistage design has attractive features such as sharing a common control group in a study that investigates multiple treatment interventions concurrently and allowing use of an adaptive design to drop arms for futility and efficacy at interim analyses. Another potential feature of a multiarm multistage design is to consider adding new treatment arm(s) to an on‐going study, known as a platform trial. Apart from the benefit of sharing the concurrent control group, adding new treatment arm(s) provides extra treatment option(s) to future patients who are yet to be enrolled to the trial. This adaptive feature also enhances future patient benefit as the newly available agents could be tested immediately in the trial when they are available. Moreover, the overall cost of studying a new treatment intervention would be reduced as the cost of setting up an independent trial can be avoided when the new agent can be tested in an on‐going trial. Examples of on‐going trials that consider the feature of adding new treatment arm(s) include STAMPEDE,^1^ I‐spy 2,^2^ and Glioblastoma (GBM) AGILE.^3^

To date, limited statistical literature on methodology for adding arms is available. Cohen et al^4^ provided a review of papers discussing methodology and practical considerations for adding arms to an on‐going clinical trial. With the decision that a newly available treatment is always added to an on‐going trial, Elm et al^5^ compared three analysis approaches based on the operating characteristics of the trial, which originally has a standard two‐arm design. Restricting a fixed maximum number of arms that can be added to a study, Ventz et al^6^ illustrated the benefits of a rolling‐arms design, which is a specific platform design that adds new arms to a study when they become available. On other aspects, Ventz et al^7^ investigated three randomisation procedures for the design that adds new arms at different time points; Wason et al^8^ investigated the impact of adding arms on family‐wise error rate. We find that the exact time to open new arm(s) has not been well addressed by these authors. The strategy of always adding in the treatments once they become available may lead to having little power for each treatment or prolonging the overall duration of a study. On the contrary, being inflexible on including new treatments may result in missing the chance of identifying a truly effective treatment.

In the context of screening a large number of agents, Yuan et al^9^ proposed a platform trial design, whereby a new treatment arm is opened once an experimental treatment graduates or is dropped. Focusing on futility monitoring, Hobbs et al^10^ proposed a framework for designing a screening platform trial that could drop arms or add new agents using Bayesian modelling. Here, we investigate the strategy of adding a new treatment to an existing study based on responses that are observed up to date. We focus on phase II/III setting where the objective of a trial is to study the efficacy of treatments. The situation we consider here is similar to those described in the works of Elm et al^5^ and Ventz et al,^6^ where a new treatment becomes available after a trial on different interventions for the same disease has commenced but prior to the end of enrollment. Instead of following a stringent strategy, we investigate when would be best to add a new treatment arm to an on‐going trial. To provide insight into how to decide whether to add a new treatment to an existing study, we consider a two‐stage multiarm trial setting. We propose a decision‐theoretic framework to provide guidance on when to add an arm to an on‐going study based on the observed treatment responses to date.

A decision‐theoretic approach explores the consequences of possible decisions and provides an optimal action that gives the highest gain on making a decision^11^, ^12^, ^13^; Hee et al^14^ provided systematic review on decision‐theoretic designs for clinical trials. The notion of our framework is similar to the work of Berry and Ho,^15^ who investigated stopping boundaries for drug development programs. However, we consider a frequentist utility instead of a Bayesian one as the common practise of clinical studies emphasises the freqentist operating characteristic of the trial. For some common diseases, where many treatment options are available, a trial might be able to identify as many effective treatments as possible. In others, being able to identify one efficacious treatment from a study would be considered a success. To cover these different prospects, we consider two types of utility in this work: one depends on the number of rejected hypotheses, the other is a binary value that equals one when at least one hypothesis is rejected by the study. The expectation of the former corresponds to the expected number of rejected hypotheses, and of latter the disjunctive power when at least one of the rejected hypotheses are false under the null. They are examples of design criteria for designing a trial that has multiple clinical objectives.^16^ A utility function reflects the point of view of a decision marker. For ease of exposition, we do not include other trial aspects such as monetary values in our illustration. This may represent the interests of an academic trial sponsor who has limited budget for recruiting more patients but want to maximise the chance of declaring a trial success by adding a new treatment arm to an on‐going trial. Other decision‐makers such as trial sponsors and health regulators can incorporate the relevant aspects into the utility function and conduct the analysis described in this work.

The structure of this paper is as follows. In Section 2, we describe the background and key elements of our decision‐theoretic framework. In Section 3, we illustrate the optimal decision that is made based on stage one responses for a case study of a trial in osteoarthritis,^17^ and the impact of prior distribution on the optimal decision. In Section 4, we illustrate the framework for an initial design that has (i) one treatment and a control arms, and (ii) two treatment and a control arms. In Section 5, we summarise our investigation, discuss the limitations of our work, and consider future work.

## 2 BACKGROUND AND DECISION‐THEORETIC FRAMEWORK

Decision theory methods provide an optimal decision to a problem by assessing the utility of each possible action. The utility is often measured in quantitative value, which is computed by subtracting the costs of taking the action from the possible gain (or loss if the outcome is negative). In this work, we consider a two‐stage setting. We aim to maximise the expected utility by making a decision at the end of stage one based on data observed up to that point. In this section, we first describe the trial setting and the distributions of the responses. We then present the analysis procedure to compute the probabilities of rejecting hypotheses based on a multivariate normal distribution prior to implementation of stage two of the trial. Following that, we specify the utility and describe the process of identifying the optimal decision, ie, to add or not to add a treatment arm to an on‐going trial based on the observed stage one responses.

### 2.1 Distribution of data

Consider a two‐stage multiarm trial that initially has *K* treatments and a control treatment, with a total sample size *N* = *N*_1_ + *N*_2_, where *N*_*s*_ is stage *s* sample size, *s* = 1,2. Denote *k* = 0 for a control treatment and *k* = 1,…,*K* for experimental treatments. Let *X*_*kj*_ be the response of treatment *k* on subject *j* = 1,…,*n*_*ks*_, where *n*_*ks*_ is the sample size of treatment *k* for stage *s*, $\sum_{k=0}^{K}n_{ks}=N_{s}$ and *n*_*k*1_ + *n*_*k*2_ = *n*_*k*_. Having observed *N*_1_ responses in stage one, we could investigate the benefit of adding a new treatment arm, denoted by *K* + 1, to the on‐going study. A decision problem is to decide whether to add a new treatment and decide the value of *N*_2_ and *n*_*k*2_, *k* = 0,…,*K* + 1 if arm *K* + 1 is added to the trial. The value of *n*_*K* + 1,2_ would depend on the randomisation procedure/scheme of the study; the value of *N*_2_ would affect the power of making inference on treatment *K* + 1. For ease of exposition, we restrict *N*_2_ to be fixed in the illustration. The case where additional patients are available if an arm is added is considered in the Discussion section. Following the decision to add an arm, we consider the use of an equal randomisation procedure such that each arm has stage two sample size *N*_2_/(*K* + 2). In other words, the decision of adding a new treatment arm would cause the initial arms to have a smaller stage‐two sample size per arm.

Consider that *X*_*kj*_ are identically and independently normally distributed with mean *μ*_*k*_ and variance $\sigma_{k}^{2}$, where $\sigma_{k}^{2}$ is a known value. Denote by ${\bar{X}}_{k.s}=\frac{\sum_{j=1}^{n_{ks}}X_{kj}}{n_{ks}}$ the stage *s* mean response of treatment *k*, ${\bar{X}}_{k}=w_{k1}{\bar{X}}_{k.1}+w_{k2}{\bar{X}}_{k.2}$ as the mean response of treatment k over both stages, $w_{ks}=\frac{n_{ks}}{n_{k}}$. We have ${\bar{X}}_{k.s}\overset{iid}{∼}N(\mu_{k},\sigma_{k}^{2}/n_{ks})$ and ${\bar{X}}_{k}∼N(\mu_{k},\sigma_{k}^{2}/n_{k})$. Without loss of generality, consider a control treatment *k* = 0 has *μ*_0_ = 0. For *k* = 1,…,*K*,*K* + 1, let *μ*_*k*_ follow a normal prior distribution with mean *m*_*k*0_ and variance *v*_*k*0_. Prior to implementing the trial, ${\bar{X}}_{k.1}$ has a prior predictive distribution that follows a normal distribution with mean *m*_*k*0_ and variance $\frac{\sigma_{k}^{2}}{n_{k1}}+v_{k0}$. Analogously, when treatment *K* + 1 is added to the trial at the end of stage one of the trial, the prior predictive distribution of ${\bar{X}}_{K+1.2}$ is a normal distribution with mean *m*_*K* + 1,0_ and variance $\frac{\sigma_{K+1}^{2}}{n_{K+1,2}}+v_{K+1,0}$.^18^

After observing stage one, the posterior distribution of *μ*_*k*_ has mean

$$m_{k1}=\frac{v_{k0}}{\frac{\sigma_{k}^{2}}{n_{k1}}+v_{k0}}{\bar{x}}_{k.1}+\frac{\frac{\sigma_{k}^{2}}{n_{k1}}}{\frac{\sigma_{k}^{2}}{n_{k1}}+v_{k0}}m_{k0}$$

and variance

$$v_{k1}={\left(\frac{1}{v_{k0}}+\frac{n_{k1}}{\sigma_{k}^{2}}\right)}^{-1},$$

which become the prior moments for *μ*_*k*_ of the next stage. The normal predictive distribution for ${\bar{X}}_{k.2}$ has mean *m*_*k*1_ and variance $\frac{\sigma_{k}^{2}}{n_{k2}}\;+\;v_{k1}$.^18^ Note that *m*_*k*1_ depends on stage one observation via ${\bar{x}}_{k.1}$. In what to follow, let $f_{{\bar{x}}_{k.1}}({\bar{x}}_{k.1})$ and $f_{{\bar{x}}_{k.2}}({\bar{x}}_{k.2}|{\bar{x}}_{k.1})$ denote the (predictive) probability density function of stage one responses and of stage two responses. They are used in the computation of expectations.

### 2.2 Hypothesis testing

We now describe the analysis plan of the trial. Without loss of generality, positive values indicate that a treatment is more effective than a control treatment on the patients. Compare treatment *k* with the control treatment *k* = 0, a hypothesis test considers

$$\begin{array}{ll} & \text{null hypothesis},H_{0k}:\;\mu_{k}-\mu_{0}\leq 0,\\ & \text{alternative hypothesis},H_{1k}:\;\mu_{k}-\mu_{0}>0. \end{array}$$

At the end of the trial, we reject a hypothesis *H*_0*k*_ when

$$\frac{\left(w_{k1}{\bar{X}}_{k.1}+w_{k2}{\bar{X}}_{k.2})-(w_{01}{\bar{X}}_{0.1}+w_{02}{\bar{X}}_{0.2}\right)}{\sqrt{\frac{\sigma_{k}^{2}}{n_{k1}+n_{k2}}+\frac{\sigma_{0}^{2}}{n_{01}+n_{02}}}}>b_{k2}.$$

When the null hypothesis is true, the left‐hand side of the first expression is normally distributed with mean zero and variance one. To get a level *α* test, we compare this test statistics to the rejection boundary, *b*_*k*2_ = Φ^−1^(1 − *α*), where Φ^−1^ denotes the cumulative density function of the standard normal distribution. In some trials with *K* > 1, it is desirable to control the family‐wise type one error rate, ie, the probability of making at least one type one error is ≤*α*, or in the strong sense that this error rate is ≤*α* for any configuration of true and false null hypotheses. Methods such as Dunnett's test, Holm‐Bonferroni method, and closed testing procedure are often used to control the error rate in multiarm multistage designs. Without loss of generality, we consider controlling the family‐wise error rate in the strong sense. We use Bonferroni correction, ie, when *K* > 1 hypotheses are tested by the study, we choose *b*_*k*2_ = Φ^−1^(1 − *α*/*K*).

Since ${\bar{X}}_{k.s}$, *k* = 0,1,…,*K*, *s* = 1,2, are independent and normally distributed, $w_{ks}{\bar{X}}_{k.s}$ are also independent and normally distributed but with a variance scaled by $w_{ks}^{2}$. It can be shown that the differences in mean response of treatments for stage two given the stage one data follow a multivariate normal distribution, ie,

$$\left(\begin{array}{c} w_{12}\;{\bar{X}}_{1.2}-w_{02}\;{\bar{X}}_{0.2}\\ w_{22}\;{\bar{X}}_{2.2}-w_{02}\;{\bar{X}}_{0.2}\\ \vdots \\ w_{K2}\;{\bar{X}}_{K.2}-w_{02}\;{\bar{X}}_{0.2} \end{array}\right)∼MVN\left[\left(\begin{array}{c} m_{11}\\ m_{21}\\ \vdots \\ m_{K1} \end{array}\right),\;\mathrm{diag}\left(w_{12}^{2}\left(\frac{\sigma_{1}^{2}}{n_{12}}+v_{11}\right),\;\text{…}\;,w_{K2}^{2}\left(\frac{\sigma_{K}^{2}}{n_{K2}}+v_{K1}\right)\right)+w_{02}^{2}\frac{\sigma_{0}^{2}}{n_{02}}{\text{1}}_{K}\right],$$

where **1**_*K*_ is a square matrix of ones with dimension *K*. We can compute the probabilities of rejecting some hypotheses by evaluating the cumulative density function of this distribution using some numerical software. For example, the probability of rejecting all hypotheses except hypothesis *r* and hypothesis *u* is

(1) $$\begin{array}{ll} & \;P(H_{0r}\;\text{and}\;H_{0u}\;\text{are not rejected},H_{0l},l\in \{1,\text{…},K\}\backslash \{r,u\}\;\text{are rejected})\\ & \;=P\left[w_{r2}\;{\bar{X}}_{r.2}-w_{02}\;{\bar{X}}_{0.2}\leq Z(r,2),\;w_{u2}\;{\bar{X}}_{u.2}-w_{02}\;{\bar{X}}_{0.2}\leq Z(u,2),\;w_{l2}\;{\bar{X}}_{l.2}-w_{02}\;{\bar{X}}_{0.2}\right.\\ & \;>\left.Z(l,2),l\in \{1,\;\text{…}\;,K\}\backslash \{r,u\}\right]\\ & \;=\int_{l_{1}}\int_{l_{2}}\int_{l_{K}}f({\bar{x}}_{2})d({\bar{x}}_{2}), \end{array}$$

where $f({\bar{x}}_{2})$ is the pdf of the multivariate normal distribution, $Z(k,2)=b_{k2}\sqrt{\frac{\sigma_{k}^{2}}{n_{k1}+n_{k2}}+\frac{\sigma_{0}^{2}}{n_{01}+n_{02}}}-(w_{k1}{\bar{X}}_{k.1}-w_{01}{\bar{X}}_{0.1})$, *k* = 1,…,*K*, and *l*_*k*_ denote the regions of the integrals; *l*_*k*_ = [*Z*(*k*,2),*∞*) for *k* ∈ {1,…,*K*}\{*r*,*u*}, and *l*_*k*_ = ( −*∞*,*Z*(*k*,2)] for *k* ∈ {*r*,*u*}. We consider this *K*‐dimensional joint distribution when we compute the expected utility of not adding an arm to an on‐going trial.

Note that nonequal *w*_*ks*_, *k* = 1,…,*K*, imply that the trial has different number of observations on different arms. This could happen for example by design, ie, using unequal treatment allocation probabilities, or because of the presence of missing observations and/or delayed outcomes. With equal randomisation probability and fully observed responses that are available at the time of making a decision, we have *w*_*ks*_ = *w*_0*s*_ for *k* = 1,…,*K*, and *s* = 1,2.

For the newly added arm, concurrent control responses are used in making inference and we reject *H*_0,*K* + 1_ when

$${\bar{X}}_{K+1.2}-{\bar{X}}_{0.2}>b_{K+1,2}\sqrt{\frac{\sigma_{K+1}^{2}}{n_{K+1,2}}+\frac{\sigma_{0}^{2}}{n_{02}}}.$$

To be consistent with the notation, we denote $Z(K+1,2)=b_{K+1,2}\sqrt{\frac{\sigma_{K+1}^{2}}{n_{K+1,2}}+\frac{\sigma_{0}^{2}}{n_{02}}}$. Note that the joint distribution of ${\bar{X}}_{K+1.2}-{\bar{X}}_{0.2}$ and the differences in mean response of *K* treatments are also a multivariate normal distribution. We use this *K* + 1‐dimensional multivariate normal distribution to compute the expected utility of adding an arm to an on‐going trial, ie,

$$\begin{array}{ll} \left(\begin{array}{c} w_{12}\;{\bar{X}}_{1.2}-w_{02}\;{\bar{X}}_{0.2}\\ \vdots \\ w_{K2}\;{\bar{X}}_{K.2}-w_{02}\;{\bar{X}}_{0.2}\\ {\bar{X}}_{K+1,.2}-{\bar{X}}_{0.2} \end{array}\right) & ∼MVN\left[\left(\begin{array}{c} m_{11}\\ \vdots \\ m_{K1}\\ m_{K+1,0} \end{array}\right),\;\mathrm{diag}\left(w_{12}^{2}\left(\frac{\sigma_{1}^{2}}{n_{12}}+v_{11}\right),\;\text{…}\;,w_{K2}^{2}\left(\frac{\sigma_{K}^{2}}{n_{K2}}+v_{K1}\right),\frac{\sigma_{K+1}^{2}}{n_{K+1,2}}+v_{K+1,0}\right)\right.\\ & \;\left.+\;\mathrm{diag}\left(w_{02}^{2}\frac{\sigma_{0}^{2}}{n_{02}},\;\text{…}\;,w_{02}^{2}\frac{\sigma_{0}^{2}}{n_{02}},\frac{\sigma_{0}^{2}}{n_{02}}\right)\right]. \end{array}$$

### 2.3 Elements of decision‐theoretic framework

We now describe the elements of our decision‐theoretic framework. Consider rejecting a null hypothesis, *H*_0*k*_, as a gain and not rejecting the hypothesis as zero loss after implementing a trial. We define the utility in the following two different ways:

- $U_{D}^{c}=k$ when *k* hypotheses are rejected, *k* = 0,1,…,*K*;
- $U_{D}^{o}=1$ when at least one hypothesis is rejected, $U_{D}^{o}=0$ otherwise.

The expectation of $U_{D}^{c}$ corresponds to the expected number of rejected hypotheses, whereas the expectation of $U_{D}^{o}$ corresponds to disjunctive power when at least one of the rejected hypotheses is false under the null. The former is an example of expectation criteria and the latter is an example of exceedance criteria that are often considered when designing a study that has multiple clinical objectives.^16^ We can find the expected values of these utilities by evaluating the probability of rejecting some number of hypotheses. For example, the probability of rejecting *K* − 2 hypothesis out of *K* hypotheses is the sum of (1) over all choices of *r* and *u*.

Within a two‐stage setting, we consider that a decision needs to be made after observing stage one of the trial, ie, the decision, *D* ∈ {*D*_*a*_,*D*_*Na*_}, where *D*_*a*_ is the decision that adds a new treatment arm to an on‐going trial, and *D*_*Na*_ is the decision that does not add a new treatment arm. The decision of adding the arm, *D*_*a*_, would reduce *n*_*k*2_, ie, stage two sample size of treatment *k*, and require adjustment of the rejection boundaries to control the prespecified family‐wise error rate. The decision of not adding the arm, *D*_*Na*_, corresponds to the initial design of the trial.

Let ${\bar{X}}_{s}$ and ${\bar{x}}_{s}$ be the vectors ${\left({\bar{X}}_{0.s},\;\text{…}\;,{\bar{X}}_{K.s}\right)}^{\prime }$ and ${\left({\bar{x}}_{0.s},\;\text{…}\;,{\bar{x}}_{K.s}\right)}^{\prime }$ for *s* = 1,2, representing data from treatment arms 0 to *K* in stages one and two. The general procedure of employing our framework is as follows. Firstly, we choose a utility, either $U_{D}^{c}$ or $U_{D}^{o}$, according to the objective of a trial. Then, we compute and compare the expected utility of each decision to identify whether it is worth adding in the new treatment arm.

To be more specific, given observed data ${\bar{x}}_{1}$ and ${\bar{x}}_{2}$ and, if we add an arm, ${\bar{x}}_{K+1.2}$, the utility $U_{D_{Na}}^{c}$ and $U_{D_{a}}^{c}$ from taking each action is given by

$$U_{D_{Na}}^{c}({\bar{x}}_{1},{\bar{x}}_{2})=\overset{K}{\underset{k=1}{\sum }}I_{\left\{w_{k2}{\bar{x}}_{k.2}-w_{02}{\bar{x}}_{0.2}>Z(k,2)\right\}}$$

and

$$U_{D_{a}}^{c}({\bar{x}}_{1},{\bar{x}}_{2},{\bar{x}}_{K+1.2})=\overset{K}{\underset{k=1}{\sum }}I_{\left\{w_{k2}{\bar{x}}_{k.2}-w_{02}{\bar{x}}_{0.2}>Z(k,2)\right\}}+I_{\left\{{\bar{x}}_{K+1.2}-{\bar{x}}_{0.2}>Z(K+1,2)\right\}},$$

which give the number of hypotheses rejected in each case. If the objective of a trial is to optimise $U_{D}^{o}$, the utility from taking each decision is given by

$$U_{D_{Na}}^{o}({\bar{x}}_{1},{\bar{x}}_{2})=\min\left\{1,U_{D_{Na}}^{c}({\bar{x}}_{1},{\bar{x}}_{2})\right\}$$

and

$$U_{D_{a}}^{o}({\bar{x}}_{1},{\bar{x}}_{2},{\bar{x}}_{K+1,2})=\min\left\{1,U_{D_{a}}^{c}({\bar{x}}_{1},{\bar{x}}_{2},{\bar{x}}_{K+1.2})\right\},$$

which equal to 1 if any hypothesis is rejected and 0 otherwise.

The expected utilities are then given by

$$E(U_{D_{Na}}\;|\;{\bar{X}}_{1}={\bar{x}}_{1})=\int \text{…}\int U_{D_{Na}}({\bar{x}}_{1},{\bar{x}}_{2})f_{{\bar{x}}_{0.2}}({\bar{x}}_{0.2}\;|\;{\bar{x}}_{0.1})\text{…}f_{{\bar{x}}_{K.2}}({\bar{x}}_{K.2}\;|\;{\bar{x}}_{K.1})d{\bar{x}}_{0.2}\text{…}d{\bar{x}}_{K.2}$$

and

$$\begin{array}{ll} E(U_{D_{a}}\;|\;{\bar{X}}_{1}={\bar{x}}_{1})=\int \text{…}\int U_{D_{a}}({\bar{x}}_{1},{\bar{x}}_{2},{\bar{x}}_{K+1.2})f_{{\bar{x}}_{0.2}}({\bar{x}}_{0.2}\;|\;{\bar{x}}_{0.1})\text{…}f_{{\bar{x}}_{K.2}}({\bar{x}}_{K.2}\;|\;{\bar{x}}_{K.1})f_{{\bar{x}}_{K+1.2}}({\bar{x}}_{K+1.2})d{\bar{x}}_{0.2}\text{…}d{\bar{x}}_{K+1.2} & \end{array}$$

for either *U* ^*c*^ or *U* ^*o*^. We identify the optimal decision such that we have

$$G_{1}({\bar{X}}_{k.1})=\max\{E(U_{D_{Na}}\;|\;{\bar{X}}_{1}={\bar{x}}_{1}),E(U_{D_{a}}\;|\;{\bar{x}}_{1}={\bar{x}}_{1})\}.$$

Note the product of the predictive densities can be shown to follow the joint distribution described previously, whereby the means of the multivariate normal distribution depend on the observed ${\bar{x}}_{k.1}$, *k* = 0,…,*K*. We could compute theses expected utilities using *pmvnorm* in *R* as they are the sums of multivariate tail areas. We use the *K* and *K* + 1‐dimensional multivariate normal distributions to compute the expected utility of not adding an arm and the expected utility of adding an arm respectively.

#### 2.3.1 The application of decision‐theoretic framework

We remind readers that, in principle, the interest of decision‐makers can be incorporated into a decision‐theoretic framework. The utilities presented earlier can be part of the utility function considered in practise. Different choices of utility function (eg, future patients' benefits), action space, and control of error rates can be formulated based on the perspective of stakeholders. Trial aspects such as trial duration and trial resources (eg, number of patients, number of trial sites) can be accounted for accordingly. For example, the utility function of trial sponsors can be a function of $U_{D}^{c}$ and monetary costs, with action space that includes decision *D*_*Na*_, decision *D*_*a*_ with extra patients and longer duration, and another option that a separate trial will be conducted for the new treatment. On the other hand, $U_{D}^{o}$ can be considered as part of the utility of public health regulators for trials in small populations such as rare diseases. By incorporating a prior distribution for the treatment effect parameters in the utility function, see, eg, the work of Berry and Ho,^15^ our framework can be extended for making an optimal decision with respect to maximising the expected profit and the number of correct rejection of hypotheses.

For ease of presentation, we illustrate the decision‐theoretic framework with $U_{D}^{c}$, $U_{D}^{o}$ and action space *D* ∈ {*D*_*a*_,*D*_*Na*_} without extra patients when a new treatment arm is opened. We do not consider monetary values and which hypotheses are true or false. We illustrate the impact of prior distribution on the optimal decision given ${\bar{x}}_{1}$ in Section 3 and investigate how the stage one responses influence the optimal decision in Section 4. Appendix B shows the results, which correspond to those in Section 2 but with extra patients.

## 3 A CASE STUDY

We first describe a completed study and illustrate how one might make the optimal decision after observing stage one of the trial. Then, we show the role of the prior distribution on the decision made.

A two‐arm randomised control trial^17^ was conducted to study the efficacy and safety/tolerability of cryoneurolysis for reduction of pain and symptoms associated with mild to moderate knee osteoarthritis. The control treatment is sham. The study considered a randomisation ratio of 2:1 in favour of the intervention, and an adaptive design that allowed for early stopping for success or futility based on the interim analyses of the primary endpoint. The primary endpoint was the mean change from baseline to Day 30 in WOMAC pain subscale score, with the hypothesis being tested that treatment (cryoneurolysis) is superior to the control treatment (sham). The effect size for sample size calculation was not reported in this paper. A maximum total sample size of 180 was determined based on results of an unpublished trial. Interim analyses were conducted when 80 patients were enrolled and every 20 patients enrolled thereafter. In other words, the minimum sample size of the study could be 80. Nevertheless, the study continued until a total of 180 patients were enrolled, with *n*_1_ = 121 and *n*_0_ = 59.

A one‐sided t‐test (lower‐tailed) with 2.5% type one error declared that the cryoneurolysis had a statistically significant greater change from baseline to Day 30 than the standard treatment. The study also found that the magnitude of the secondary outcomes far exceeded the established minimal clinically important improvement thresholds. Table II^17^ shows the mean change from baseline to Day 30 is ${\bar{x}}_{1}=$ −16.65 with estimated $\sigma_{1}^{2}/n_{1}=1.26^{2}$ for the treatment (cryoneurolysis), and ${\bar{x}}_{0}=$ −9.54 with estimated $\sigma_{0}^{2}/n_{0}=1.63^{2}$ for the standard treatment (sham). To be coherent with the exposition, we assume that the estimates are the true variances and use a one‐sided (upper‐tailed) Wald‐test with 2.5% type one error/family‐wise error rate in the illustration. Hence, we consider positive magnitudes instead of negative values to indicate effective mean change. We consider a hypothetical situation that the study has only one interim analysis and we have the option to add a new treatment arm to the study. Assume at stage one, ie, the first interim analysis when 80 patients were enrolled (with *n*_01_ = 27 and *n*_11_ = 53), we observed 8 and 5 for ${\bar{X}}_{1.1}$ and ${\bar{X}}_{0.1}$. These numbers are chosen for illustration to reflect the fact that the study did not stop for efficacy at the first interim analysis. Following the decision of adding an arm, *D*_*a*_, we consider equal randomisation probability, ie, {*n*_02_,*n*_12_,*n*_22_} = {33,33,34} and control for the family‐wise error rate. We compare this decision to the decision of not adding an arm, *D*_*Na*_, where {*n*_02_,*n*_12_,*n*_22_} = {32,68,0} and the type one error rate is controlled.

### 3.1 Expected gain of not adding an arm given stage one data

Condition on stage one mean response of treatments, the expected gain following *D*_*Na*_ is

(2) $$\begin{array}{ll} & \;E(U_{D_{Na}}\;|\;{\bar{X}}_{0.1}=5,{\bar{X}}_{1.1}=8,n_{k2}=N_{2}/2)\\ & \;=0\times P(H_{01}\;\text{is not rejected}\;|\;{\bar{X}}_{0.1}=5,{\bar{X}}_{1.1}=8,n_{02}=32,n_{12}=68)\\ & \;\;+1\times P(H_{01}\;\text{is rejected}\;|\;{\bar{X}}_{0.1}=5,{\bar{X}}_{1.1}=8,n_{02}=32,n_{12}=68)\\ & \;=P(H_{01}\;\text{is rejected}\;|\;{\bar{X}}_{0.1}=5,{\bar{X}}_{1.1}=8,n_{02}=32,n_{12}=68)\\ & \;=P({\bar{X}}_{1.2}-{\bar{X}}_{0.2}>Z(1,2)\;|\;{\bar{X}}_{0.1}=5,{\bar{X}}_{1.1}=8,n_{02}=32,n_{12}=68)\\ & \;=\int_{Z(1,2)}^{\infty }f({\bar{x}}_{1.2}-{\bar{x}}_{0.2})d({\bar{x}}_{1.2}-{\bar{x}}_{0.2}), \end{array}$$

where $f({\bar{x}}_{1.2}-{\bar{x}}_{0.2})$ is the pdf of

(3) $$\begin{array}{ll} {\bar{X}}_{1.2}-{\bar{X}}_{0.2}∼N\left(m_{11},\frac{\sigma_{1}^{2}}{n_{12}}+v_{11}+\frac{\sigma_{0}^{2}}{n_{02}}\right), & \end{array}$$

and $Z(1,2)=b_{12}\sqrt{1.26^{2}+1.63^{2}}-(\frac{53}{121}\times 8-\frac{27}{59}\times 5)$ and *b*_12_ = 1.96. We note that *m*_11_ and *v*_11_ are posterior mean and variance that depend on stage one responses (see Section 2.1). In this simple case, $U_{D_{Na}}^{o}$ and $U_{D_{Na}}^{c}$ have expectation equals to (2).

### 3.2 Expected gain of adding an arm, $E(U_{D_{a}})$, given stage one data

If the study aims to maximise the number of rejected hypotheses, the expected gain following *D*_*a*_ given stage one responses is

(4) $$\begin{array}{ll} & \;E\left(U_{D_{a}}^{c}\;|\;{\bar{X}}_{0.1}=5,{\bar{X}}_{1.1}=8,n_{k2}=N_{2}/3\right)\\ & \;=P\left({\bar{X}}_{1.2}-{\bar{X}}_{0.2}>Z(1,2),{\bar{X}}_{2.2}-{\bar{X}}_{0.2}\leq Z(2,2)\;|\;{\bar{X}}_{0.1}=5,{\bar{X}}_{1.1}=8,n_{02}=33,n_{12}=33,n_{22}=34\right)\\ & \;\;+P\left({\bar{X}}_{1.2}-{\bar{X}}_{0.2}\leq Z(1,2),{\bar{X}}_{2.2}-{\bar{X}}_{0.2}>Z(2,2)\;|\;{\bar{X}}_{0.1}=5,{\bar{X}}_{1.1}=8,n_{02}=33,n_{12}=33,n_{22}=34\right)\\ & \;\;+2\;P\left({\bar{X}}_{1.2}-{\bar{X}}_{0.2}>Z(1,2),{\bar{X}}_{2.2}-{\bar{X}}_{0.2}>Z(2,2)\;|\;{\bar{X}}_{0.1}=5,{\bar{X}}_{1.1}=8,n_{02}=33,n_{12}=33,n_{22}=34\right). \end{array}$$

If the trial considers declaring a success when at least one hypothesis is rejected, the expected gain following *D*_*a*_ is

$$\begin{array}{ll} & \;E\left(U_{D_{a}}^{o}\;|\;{\bar{X}}_{0.1}=5,{\bar{X}}_{1.1}=8,n_{k2}=N_{2}/3\right)\\ & \;=P\left({\bar{X}}_{1.2}-{\bar{X}}_{0.2}>Z(1,2),{\bar{X}}_{2.2}-{\bar{X}}_{0.2}\leq Z(2,2)\;|\;{\bar{X}}_{0.1}=5,{\bar{X}}_{1.1}=8,n_{02}=33,n_{12}=33,n_{22}=34\right)\\ & \;\;+P\left({\bar{X}}_{1.2}-{\bar{X}}_{0.2}\leq Z(1,2),{\bar{X}}_{2.2}-{\bar{X}}_{0.2}>Z(2,2)\;|\;{\bar{X}}_{0.1}=5,{\bar{X}}_{1.1}=8,n_{02}=33,n_{12}=33,n_{22}=34\right)\\ & \;\;+P\left({\bar{X}}_{1.2}-{\bar{X}}_{0.2}>Z(1,2),{\bar{X}}_{2.2}-{\bar{X}}_{0.2}>Z(2,2)\;|\;{\bar{X}}_{0.1}=5,{\bar{X}}_{1.1}=8,n_{02}=33,n_{12}=33,n_{22}=34\right). \end{array}$$

These conditional probabilities can be computed by considering the joint distribution

$$\left(\begin{array}{c} {\bar{X}}_{1.2}-{\bar{X}}_{0.2}\\ {\bar{X}}_{2.2}-{\bar{X}}_{0.2} \end{array}\right)∼MVN\left[\left(\begin{array}{c} m_{11}\\ m_{20} \end{array}\right),\left(\begin{array}{cc} \frac{\sigma_{1}^{2}}{n_{12}}+v_{11}+\frac{\sigma_{0}^{2}}{n_{02}} & \frac{\sigma_{0}^{2}}{n_{02}}\\ \frac{\sigma_{0}^{2}}{n_{02}} & \frac{\sigma_{2}^{2}}{n_{22}}+v_{20}+\frac{\sigma_{0}^{2}}{n_{02}} \end{array}\right)\right].$$

We note that *m*_11_ and *v*_11_ are the same under both decisions but the marginal distribution of $\left({\bar{X}}_{1.2}-{\bar{X}}_{0.2}\right)$ from this bivariate normal distribution is different to (3) in terms of *n*_12_ and *n*_02_. Moreover, *Z*(1,2) of *D*_*a*_ has a rejection boundary *b*_12_ = 2.24, and $Z(2,2)=2.24\sqrt{\frac{\sigma_{2}^{2}}{n_{22}}+\frac{\sigma_{0}^{2}}{n_{02}}}$.

### 3.3 The role of prior moments

We fix *σ*_2_ = *σ*_1_ and investigate the impact of prior moments on the optimal decision for this case study. For a choice of {*m*_10_,*m*_20_,*v*_10_,*v*_20_}, we compute the expected utility for each decision and identify the optimal decision for this particular setting. For example, fix {*m*_10_,*m*_20_,*v*_10_,*v*_20_} = {0,5,10,10}, we get $E(U_{D_{Na}}|\;{\bar{X}}_{0.1}=5,{\bar{X}}_{1.1}=8,n_{02}=32,n_{12}=68)=0.63$. If the trial objective is to maximise the number of rejected hypotheses, we get $E(U_{D_{a}}^{c}|\;{\bar{X}}_{0.1}=5,{\bar{X}}_{1.1}=8,n_{02}=33,n_{12}=33,n_{22}=34)=0.815$, and hence *D*_*a*_ is the optimal decision as this decision would have (0.815/0.63 − 1) × 100 = 29% chance of rejecting an extra hypothesis. If the trial objective is to maximise $U_{D}^{o}$, not adding an arm is the optimal decision for this choice of prior moments as we get $E(U_{D_{a}}^{o}|\;{\bar{X}}_{0.1}=5,{\bar{X}}_{1.1}=8,n_{02}=33,n_{12}=33,n_{22}=34)=0.618$, which is smaller than 0.63.

**Figure 1:**
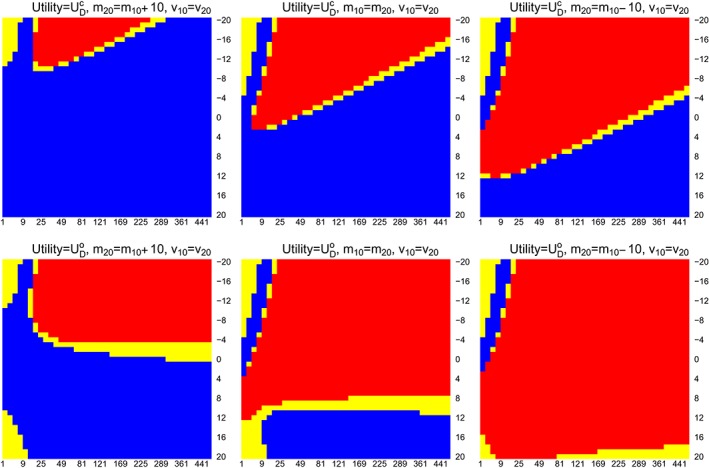
Optimal decisions: blue indicates adding an arm, red indicates do not add arm. The yellow region indicates $0<|E(U_{D_{a}}|\cdot )-E(U_{D_{Na}}|\cdot )|<0.01$. First and second row correspond to using $U_{D}^{c}$ and $U_{D}^{o}$ in the framework, x‐axis and y‐axis correspond to the value of v_10_ and m_10_ [Colour figure can be viewed at wileyonlinelibrary.com]

We repeat this process for different {*m*_10_,*m*_20_,*v*_10_,*v*_20_} and illustrate the optimal decisions on Figure 1. On each plot, *x*‐axis and *y*‐axis correspond to the value of *v*_10_ and *m*_10_, blue and red indicate adding an arm and not adding an arm are the optimal decision for a choice of *v*_10_ and *m*_10_. The yellow region indicates $0<|E(U_{D_{a}}|\cdot )-E(U_{D_{Na}}|\cdot )|<0.01$, which could indicate indifference between the two decisions as the difference between the two expected utility is less than 0.01, or *D*_*Na*_ is better from monetary and logistics point of view. We include this region in the analysis to account for the presence of Monte Carlo error when using *pmvnorm* on *R* to evaluate the probabilities. The first and the second row of plots correspond to using $U_{D}^{c}$ and $U_{D}^{o}$, respectively, in the framework. Each column corresponds to the situation that *m*_10_/*m*_20_ < 1, *m*_10_/*m*_20_ = 1, and *m*_10_/*m*_20_ > 1.

We see that the choices of prior moments have some impact on the optimal decision. When $U_{D}^{c}$ is considered in the decision‐theoretic framework, choosing positive prior means and large prior variances would generally lead to adding an arm as the optimal decision. On the other hand, when $U_{D}^{o}$ is the objective of the study, it is better not to add the new treatment arm (when *m*_10_ > *m*_20_), unless both the prior means and variances are very large. This analysis highlights the fact that it is more beneficial to add a new treatment arm to an on‐going trial when the experimenters believe that the new treatment is considerably more efficacious than the control treatment and/or the initial treatment. Note that, when *m*_10_ < <0, $E(U_{D_{Na}}\;|\;{\bar{X}}_{0.1}=5,{\bar{X}}_{1.1}=8,n_{k2}=N_{2}/2)$ is very close to zero and hence *D*_*a*_ is the optimal decision on the top left corner in the plots. In practise, consider prior means < < 0 for studies where positive magnitudes indicate that better treatment effect is not logical. This is because, if a treatment is believe to have negative treatment effect, it will not be chosen to be tested in a phase II or phase III clinical trial. We include this part in the illustration to remind users who might conduct such an analysis for studies that consider negative magnitudes as efficacious.

## 4 RELATIONSHIP BETWEEN STAGE ONE RESPONSES AND OPTIMAL DECISION

We now explore how the optimal decision depends on the stage one data. Having chosen the utility and the values of {*m*_10_,*m*_20_,*v*_10_,*v*_20_}, we can identify the optimal decisions for a range of stage one mean response of treatments. We assume equal treatment randomisation probability for both stages, type one/family‐wise error rate of 5%, and Bonferroni correction when more than one experimental treatment is included in the trial. In what follows, we denote an optimal decision by *D* ^∗^.

The first illustration considers an initial design that has one treatment arm and a control arm, whereas the second illustration has two treatment arms and a control arm. Without loss of generality, we depict the framework with a moderate and a large total sample size, respectively, in the examples.

### 4.1 Initial design: one treatment and a control arms

Consider a trial that has one treatment arm (*k* = 1) and a control arm (*k* = 0) initially, and stage‐wise sample sizes *N*_1_ = 400 and *N*_2_ = 300. After observing stage one responses where *n*_01_ = *n*_11_ = 200, a decision must be made, to either choose *D*_*a*_ that adds a new treatment arm (*k* = 2) to the concurrent trial, or *D*_*Na*_ that do not add the new treatment arm. Following *D*_*a*_, there will be three arms at stage two. Each arm will have *n*_*k*2_ = *N*_2_/3 = 100, for *k* = 0,1,2 (two treatments and a control). The family‐wise error rate is controlled as two hypotheses will be tested at the end of the trial. We compute $E(U_{D_{a}}^{c}|\;{\bar{X}}_{0.1},{\bar{X}}_{1.1},n_{k2}=N_{2}/3)$ and $E(U_{D_{a}}^{o}|\;{\bar{X}}_{0.1},{\bar{X}}_{1.1},n_{k2}=N_{2}/3)$ using *pmvnorm* on *R* as we did in Section 3.2 but with different values of ${\bar{x}}_{0.1},{\bar{x}}_{1.1}$ and sample sizes. When the trial follows *D*_*Na*_, the two original arms will have *n*_02_ = *n*_12_ = *N*_2_/2 = 150, and the type one error rate is controlled for a treatment effect comparison between treatment *k* = 1 and control treatment *k* = 0. We can likewise compute $E(U_{D_{Na}}|\;{\bar{X}}_{k.1},k=0,1,n_{k2}=N_{2}/2)$, and then compare this value to the expected utility of adding an arm to identify the optimal decision for given values of ${\bar{x}}_{0.1},{\bar{x}}_{1.1}$.

In general, when the objective of a trial is to maximise the number of rejected hypotheses, we find that it is more beneficial to add a new treatment arm *k* = 2 when ${\bar{x}}_{1.1}-{\bar{x}}_{0.1}$ is very large (indicating that treatment *k* = 1 is considerably better than the control treatment), or when ${\bar{x}}_{1.1}-{\bar{x}}_{0.1}$ is very small (indicating that treatment *k* = 1 is considerably worse than the control treatment). This could represent two extreme cases. (i) When we have enough power for testing *H*_01_, it is more beneficial to spend the remaining resources on testing the new treatment *k* = 2. (ii) When we observe that the current treatment is not efficacious at all, we shall not spend all of the remaining resources on that treatment arm but open a new arm for studying another intervention in the on‐going trial. On the other hand, for the trials that would consider a success when at least one hypothesis is rejected, the benefit of adding a new treatment arm to an on‐going trial is substantial when the initial treatment is not effective. In the rare scenario where a promising finding is shown by stage one responses, adding a new treatment arm only results in negligible improvement to the utility of the trial. This is because the maximum of $U_{D}^{o}$ is one and rejecting one hypothesis with high probability is sufficient to declare a success.

**Figure 2:**
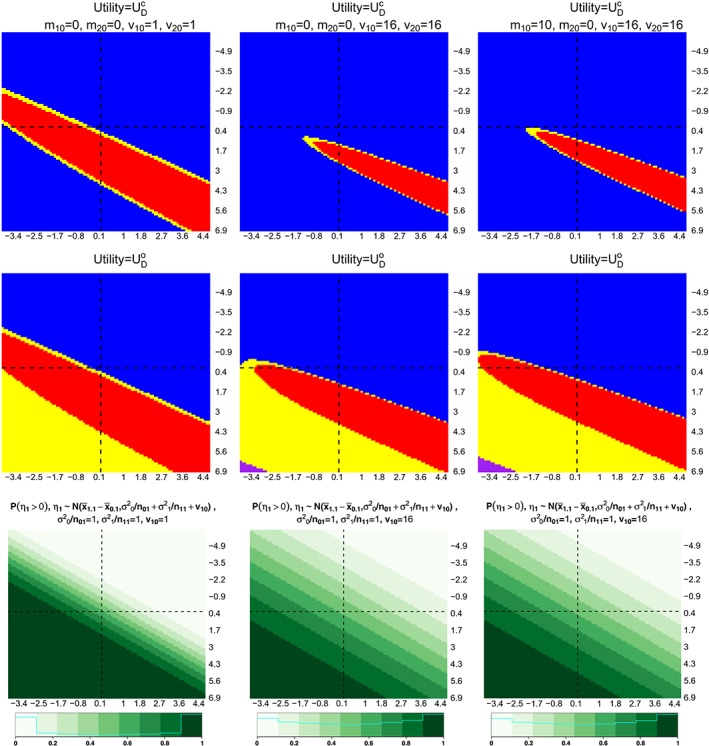
Optimal decisions when $U_{D}^{c}$ (first row) and $U_{D}^{o}$ (second row) are considered in the framework. Last row shows the probabilities that ${\bar{x}}_{1.1}-{\bar{x}}_{0.1}>0$. ${\bar{x}}_{0.1}$ is plotted on x‐axis and ${\bar{x}}_{1.1}$ on y‐axis, dashed lines are plotted at approximately zero. Each column considers a choice of {m_10_, m_20_, v_10_, v_20_}. Blue and red indicate adding an arm and not adding an arm are the optimal decision. Purple and yellow indicate that both decisions have the same expected utility and $0<|E(U_{D_{a}}|\cdot )-E(U_{D_{Na}}|\cdot )|<0.01$ [Colour figure can be viewed at wileyonlinelibrary.com]

Figure 2 shows the optimal decisions for different pairs of mean response of treatments, where ${\bar{x}}_{0.1}$ is plotted on the *x*‐axis, and ${\bar{x}}_{1.1}$ on the *y*‐axis. Each column corresponds to using different values of {*m*_10_, *m*_20_, *v*_10_, *v*_20_} in the framework. The last row of plots in the figure shows the probabilities that ${\bar{x}}_{1.1}-{\bar{x}}_{0.1}>0$. For ease of comparison, we focus on the same values of ${\bar{x}}_{0.1}$ and of ${\bar{x}}_{1.1}$ for different {*m*_10_, *m*_20_, *v*_10_, *v*_20_} in Figure 2. The interpretation of a plot on the first two rows in Figure 2 is as follows. For a pair of ${\bar{x}}_{0.1}$ and ${\bar{x}}_{1.1}$, the optimal decision is represented by a colour code: blue indicates *D*_*a*_ has a larger utility value given stage one data, red indicates *D*_*Na*_ has a larger utility value given stage one data. The yellow regions in the plots indicate $0<|E(U_{D_{a}}|\cdot )-E(U_{D_{Na}}|\cdot )|<0.01$, whereas the purple regions indicate both decisions have the same expected utility. Both of this yellow and purple region could be interpreted as indifference between the two decisions as the gain is less than 0.01, or *D*_*Na*_ is better from monetary and logistics point of view.

The blue region on the second quadrant (top right) in all plots highlights that *D*_*a*_ is the optimal decision when the initial treatment is not efficacious (as indicated by the light green region on the probability plots). We see that the region for which *D*_*Na*_ is the optimal decision depend on the prior moments for *μ*_*k*_. Consider choosing $U_{D}^{c}$ as the utility in the decision‐theoretic framework. As the prior variances, *v*_*k*0_ increases, ie, when comparing (1,1) and (1,2) plots in Figure 2, the red area (indicates *D*_*Na*_ is the optimal decision) becomes smaller. This result is not surprising as the expected utility gain of having two rejection of hypotheses is dominating, ie, given *Z*(*k*,2), the relationship between variance parameters (that depend on *v*_*k*0_) and the last probability in (4) are monotonic increasing. When *m*_10_ increases, ie, treatment *k* = 1 is believed to have a positive mean response, the red area is slightly enlarged and shifted to the left and upward when we compare (1,2) with (1,3) plots. This result is less intuitive and we conjecture that the larger sample size per treatment arm (of stage two) and the rejection boundary following *D*_*Na*_ contributes to the small increase in the area of the red region. We find a similar pattern when $U_{D}^{o}$ is considered as the utility gain in the decision‐theoretic framework. In addition to that, there is a purple region where both decision *D*_*a*_ and *D*_*Na*_ give the same utility value. Within these ranges of ${\bar{x}}_{0.1}$ and ${\bar{x}}_{1.1}$, we only see the purple region in (2,2) and (2,3) plot in Figure 2. This occurs in the lower left quadrant (second row of plots), where both the expected value of $U_{D_{Na}}^{o}$ and of $U_{D_{a}}^{o}$ are approximately equal to one, and the difference between these values is trivial.

### 4.2 Initial design: two treatment and a control arm

We now illustrate the decision‐theoretic framework for a two‐stage trial that has two treatment arms, *k* = 1,2, and a control arm (*k* = 0) at stage one with *n*_*k*1_ = *N*_1_/3 = 400, and stage‐wise samples *N*_1_ = 1200 and *N*_2_ = 1800. At the end of stage one, we want to identify the best decision by considering the expected gains prior to implementation of stage two. With either decision, we control the family‐wise error rate accordingly at 5%. The decision of not adding an arm would have *n*_*k*2_ = *N*_2_/3 = 600 for *k* = 0,1,2. We can compute the expected utilities of *D*_*Na*_ with the probability as described in Section 3.2 but conditioned on ${\bar{x}}_{k.1},k=0,1,2$ and different sample sizes.

On the other hand, the decision *D*_*a*_ would involve adding in a third experimental arm, and that *n*_*k*2_ = *N*_2_/4 = 450, for *k* = 0,1,2,3. To find the expected gain of adding a new treatment arm, we need to consider possible combinations of rejecting three hypotheses, *H*_01_, *H*_02_, and *H*_03_, which might be rejected at the end of the trial. In order words, for the trials that focus on maximising the number of rejected hypotheses, we evaluate

$$E\left(U_{D_{a}}^{c}|\;{\bar{X}}_{k.1},k=0,1,2,n_{k2}=N_{2}/4\right)=\overset{3}{\underset{v=1}{\sum }}v\times P(\text{reject exactly}\;v\;\text{hypotheses out of three hypotheses});$$

for the other type of utility, we compute

$$\begin{array}{ll} & \;E\left(U_{D_{a}}^{o}|\;{\bar{X}}_{k.1},k=0,1,2,n_{k2}=N_{2}/4\right)\\ & \;=\overset{3}{\underset{v=1}{\sum }}P(\text{reject exactly}\;v\;\text{hypotheses out of three hypotheses})\\ & \;=1-P(\text{not reject all three hypotheses}), \end{array}$$

which is equivalent to the disjunctive power when at least one of the rejected hypotheses is false under the null.

We can compute these probabilities using *pmvnorm* on *R*; with a bivariate normal distribution for the expected utility of *D*_*Na*_, and a trivariate normal distribution for the expected utility of *D*_*a*_. Note that the means and variances of both multivariate normal distribution depend on the prior moments and stage one mean response of treatments. To be more specific, we have three mean response of treatments, ie, ${\bar{x}}_{0.1}$, ${\bar{x}}_{1.1}$, and ${\bar{x}}_{2.1}$, at the end of stage one of the trial. Given these values and a choice of {*m*_10_, *m*_20_, *m*_30_, *v*_10_, *v*_20_, *v*_30_}, we can compute and compare the expected utility of each decision, with either $U_{D}^{c}$ or $U_{D}^{o}$ in the framework, to identify the optimal decision. We can repeat this procedure for different ${\bar{x}}_{0.1}$, ${\bar{x}}_{1.1}$, and ${\bar{x}}_{2.1}$ for the investigation before seeing stage one of the trial.

**Figure 3:**
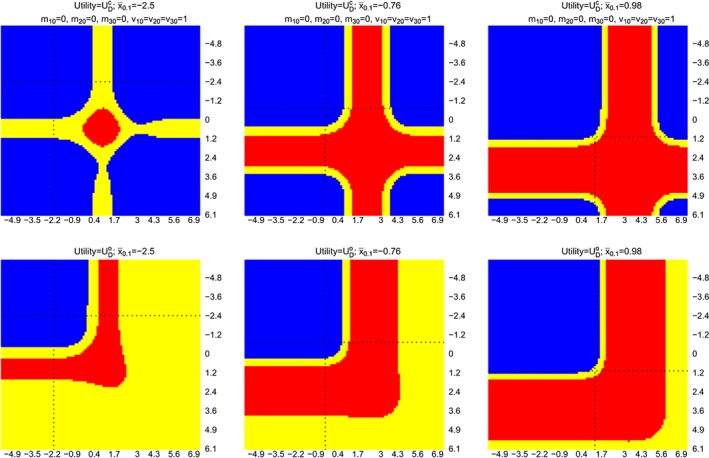
Optimal decisions when $U_{D}^{c}$ (first row) and $U_{D}^{o}$ (second row) are considered in the framework. ${\bar{x}}_{2.1}$ is plotted on x‐axis and ${\bar{x}}_{1.1}$ is plotted on y‐axis, dashed lines are plotted at approximately zero, and dotted lines correspond to the value of a ${\bar{x}}_{0.1}$. Each column considers a value of ${\bar{x}}_{0.1}$. Blue and red indicate that adding an arm and not adding an arm are the optimal decisions. Yellow indicates $0<|E(U_{D_{a}}|\cdot )-E(U_{D_{Na}}|\cdot )|<0.01$ [Colour figure can be viewed at wileyonlinelibrary.com]

Figure 3 shows the results for this multiarm trial setting. Within each plot, ${\bar{x}}_{2.1}$ is plotted on the *x*‐axis and ${\bar{x}}_{1.1}$ is plotted on the *y*‐axis, dotted line corresponds to a value of the observed ${\bar{x}}_{0.1}$. Each column corresponds to conditioning on a single ${\bar{x}}_{0.1}$; the first and second row of plots correspond to choosing $U_{D}^{c}$ and $U_{D}^{o}$, respectively, in the framework. We see that there is a trend as we move from negative to positive value of ${\bar{x}}_{0.1}$. Blue, red, and yellow indicate adding an arm, not adding an arm, and indifference between the decisions when $0<|E(U_{D_{a}}|\cdot )-E(U_{D_{Na}}|\cdot )|<0.01$ (or *D*_*Na*_ is better from monetary and logistics point of view).

The first quadrant (upper left quadrant that is divided by the dotted lines) on each plot corresponds to the situations that neither treatment k = 1 nor treatment k = 2 is better than the control treatment. The analysis shows that adding a new treatment k = 3 to the existing trial is more beneficial than continuing the trial according to the initial design, when either $U_{D}^{c}$ or $U_{D}^{o}$ is considered in the framework in this case.

When either (initial) treatment is better than the control arm (the second or the third quadrant), the framework with $U_{D}^{c}$ shows that the expected number of rejected hypotheses could be increased by adding a new treatment arm when information for the initial arms is sufficient to claim the promising finding. On the other hand, the framework with $U_{D}^{o}$ would not add a new treatment arm in this case as the promising finding on any initial treatments would fulfil the trial objectives.

When both initial treatments are more efficacious than the control treatment, adding a new treatment arm could increase the expected number of rejected hypotheses provided that the initial treatments have sufficient information to claim the promising finding. This is reflected on the first row of plots, bottom right quadrant that is divided by the dotted lines. On the other hand, for a trial that focuses on declaring a success when at least one hypothesis is rejected, the bottom right quadrant in the second row of plots shows mainly red (*D*_*Na*_ is the optimal decision) and yellow (indifference or *D*_*Na*_ is better from monetary and logistics point of view). This is because, if we have observed promising findings on both initial treatment arms, the expected value of $U_{D_{Na}}^{o}$ is already close to one. Hence, the disjunctive power can no longer be increased by testing an extra hypothesis. These findings are consistent to the findings of the simpler study that initially has two arms (one treatment and a control).

## 5 DISCUSSION

The development of treatments for diseases is a long process. It is often the case that potential treatments are not available at the same time for conducting phase II/III studies. This work highlights the benefits of adding a new treatment arm to an existing study at an interim analysis. Instead of adding in a new treatment with certainty, we propose a decision‐theoretic framework for studying when it is optimal to add an arm to an existing multiarm trial. We defined utility according to a design objective of a study, ie, (i) maximise the number of rejected hypotheses, and (ii) consider a success when at least one hypothesis is rejected. The expected value of these utilities is examples of expectation criteria and exceedance criteria, respectively. We consider a study design that initially has *K* experimental treatments and a control treatment. Having observed stage one of the trial, we identify the optimal decision by comparing the expected utility of using this initial design to the expected utility of using another design that has *K* + 2 arms at the second stage. Within the two‐stage setting, we looked at the costs where adding in a new arm would reduce stage‐two sample size per treatment arm and require adjustment to account for multiplicity. We acknowledge that multiplicity corrections might not be made in some multiarm trials. In this case, we only need to consider the cost of having smaller sample size per treatment arm and do the analysis of adding an arm analogously.

We presented how to investigate whether it is worth adding in a new treatment arm to an on‐going multiarm trial based on the observed stage one mean response of treatments for a case study. We showed the impact of prior distributions on the optimal decisions. We also illustrated the analysis before the implementation of the trial. For the trial with more initial arms, the investigation can proceed analogously as described earlier. We find that, in a multiarm study, when none of the experimental treatments are more efficacious than the control treatment, adding in a new treatment arm to the on‐going study is more beneficial than continuing the trial according to the initial design. This is because the newly added arm might demonstrate promising finding and hence increase the utility of the study. On the contrary, when the initial experimental treatments are considerably more efficacious than the control treatment, adding in a new treatment arm could potentially increase the expected number of rejected hypotheses. However, for multiarm studies that focus on disjunctive power, the decision to add a new treatment arm in this case is less substantial. This is because being able to claim any of the initial treatments as promising would have already fulfilled the study objective.

The aim of this manuscript is to present the notion of our decision‐theoretic framework and the procedure of the investigation. We remind readers that the results of the illustrations presented in this paper are not comprehensive. For ease of exposition, we do not include the consideration of other aspects in the framework, eg, monetary cost of opening the new treatment arm. We also do not compare the utility of conducting a separate trial for the new arm when it was not added to the existing trial. Practitioners can formulate their utility and action spaces accordingly, as described in Section 2.3. Nevertheless, we have not considered choosing *N*_2_ based on stage one observations in this work. A simple approach for optimising *N*_2_ at the same time could be specifying values for a cost per patient and reward for success. Together with early stopping for futility and efficacy rules, future work could investigate how best to use the trial resources by constructing a different utility function. Without adding a prior distribution for the parameter of treatment effect, future work may also incorporate the probability of finding the treatment to be significant into the utility function. This could reflect that we are indirectly favouring treatments with higher effects without considering whether the null hypotheses are true or false.

We note that, as we consider a two‐stage design whereby there is a possible change on total sample size per treatment arm, the type one error rate might be inflated. One could estimate the type one error rate, for example, by considering a null distribution for the responses and assume a prior distribution for the decision marking, then approximate the type one error. This investigation could show the magnitude of the inflation, and the experimenters could consider the significance of this inflation when making the decision for implementation. Alternatively, we could avoid this issue by replacing the aforementioned inference procedure with a combination test^19^ and conduct the investigation on adding arm adaptation in a similar fashion. Future work could consider using a recursive combination test^20^ or a group sequential approach to adjust the final test^21^, ^22^ in a decision‐theoretic framework for trials that have multiarm multistage settings.

**Figure B1:**
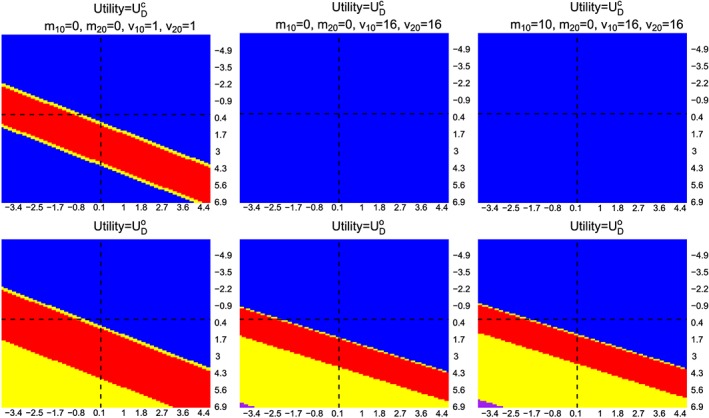
Optimal decisions when $U_{D}^{c}$ (first row) and $U_{D}^{o}$ (second row) are considered in the framework. Last row shows the probabilities that ${\bar{x}}_{1.1}-{\bar{x}}_{0.1}>0$. ${\bar{x}}_{0.1}$ is plotted on x‐axis and ${\bar{x}}_{1.1}$ on y‐axis, dashed lines are plotted at approximately zero. Each column considers a choice of {m_10_, m_20_, v_10_, v_20_}. Blue and red indicate adding an arm and not adding an arm are the optimal decision. Purple and yellow indicate that both decisions have the same expected utility and $0<|E(U_{D_{a}}|\cdot )-E(U_{D_{Na}}|\cdot )|<0.01$ [Colour figure can be viewed at wileyonlinelibrary.com]

**Figure B2:**
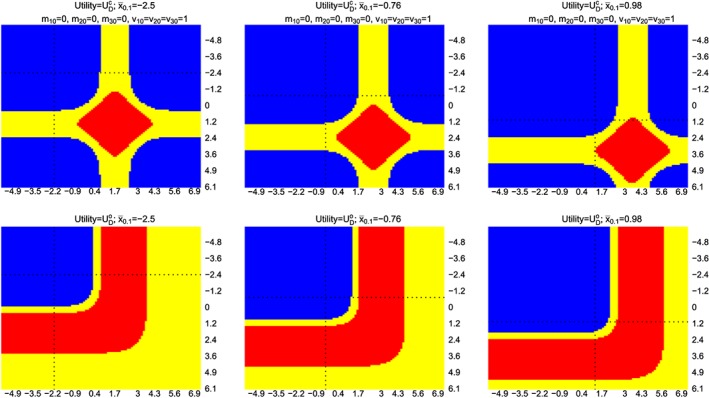
Optimal decisions when $U_{D}^{c}$ (first row) and $U_{D}^{o}$ (second row) are considered in the framework. ${\bar{x}}_{2.1}$ is plotted on x‐axis and ${\bar{x}}_{1.1}$ is plotted on y‐axis, dashed lines are plotted at approximately zero, and dotted lines correspond to the value of a ${\bar{x}}_{0.1}$. Each column considers a value of ${\bar{x}}_{0.1}$. Blue and red indicate that adding an arm and not adding an arm are the optimal decisions. Yellow indicates $0<|E(U_{D_{a}}|\cdot )-E(U_{D_{Na}}|\cdot )|<0.01$ [Colour figure can be viewed at wileyonlinelibrary.com]

We acknowledge that more patients may be recruited when a new arm is added to an existing trial. For clinical trials taking place on small populations, such as for rare diseases, targeted subgroups, and paediatrics, recruiting more patients can be infeasible and/or time consuming. Figures 2 and 3 show the decision of adding arm when the on‐going trial has fixed resources and time. Figures B1 and B2 in Appendix B show the optimal decisions when extra patients are available. Table B1 shows the number of patients considered in these illustrations. We find that the pattern of the corresponding plots is similar when comparing the trials that have extra patients with the trials without any extra patients. The designs that initially has two arms and which aims to maximise the number of rejected hypotheses, $U_{D}^{c}$, are exceptional cases. For these cases, ie, when prior means ≥0 and prior variances are large (correspond to (1,2) and (1,3) plots in Figure B1), we find that adding the new treatment arm is the optimal decision given those observed stage one mean responses. This finding is not surprising as the initial trial resources are not affected by the decision and adding in the new arm increases the chance of rejecting an extra hypothesis.

Here, we have focused on a normal outcome. To our knowledge, many clinical trials in psychiatry and psychology studies involve normally distributed outcomes. The systematic review by Wason et al^23^ considered 59 multiarm trials, of which 22 (37%) have continuous primary outcome. We think it is possible to amend such a decision‐theoretic framework for trials with a binary outcome. This can be done by changing the test statistics and making some assumptions about the variances of the response probabilities. For other types of outcome, the framework can be applied when the trial sample sizes are large. This is because the test statistics can be approximated by the normal distribution even if the individual outcome data is not normal. We acknowledge that most of the trials in practise have several secondary outcomes that it may be important to consider, or involve subgroup analysis and follow‐up measurements. Further investigation is required to account for these. Besides that, we have not facilitated the standard power and sample size calculation in the framework. Wason et al^24^ investigated sample size for a multiarm multistage drop‐the‐losers design. Future work could consider the impact of adding an arm on this design, different approaches and perspectives for the sample size calculation,^25^, ^26^, ^27^, ^28^, ^29^, ^30^, ^31^, ^32^ and also account for the presence of missing responses. Some authors^33^, ^34^ have tackled the presence of missing responses from the perspective of design of experiments, by having more subjects enrolled to the arm that is expected to have higher missing response rate. Other future work could incorporate stage one control responses when making pair‐wise comparison between the newly added treatment and the control treatment. A caveat on this aspect is that there might be population drift if the overall duration of a trial is long. One could also consider the impact of using different randomisation strategies such as a response adaptive randomisation procedure that is robust to time trend.^35^ We note that we have not focused on parameter estimation in this work. In seamless phase II/III clinical trials with early stopping for futility, methods have been proposed for unbiased estimation.^36^, ^37^, ^38^ Stallard and Kimani^39^ indicated that (conditionally) unbiased estimators for the trial that adds arm(s) could be complicated to derive. This highlights the fact that more research on adding arm(s) to an on‐going trial is required.

## APPENDIX A

### A.1 THE DERIVATION OF EXPECTED GAIN OF ADDING AN ARM

This section illustrates the intermediate steps of obtaining the expected utilities in Section 3.2, ie,

$$\begin{array}{ll} & \;E\left(U_{D_{a}}^{c}|{\bar{X}}_{0.1}=5,{\bar{X}}_{1.1}=8,n_{k2}=N_{2}/3\right)\\ & \;=0\times P(H_{01}\;\text{and}\;H_{02}\;\text{are not rejected}\;|\;{\bar{X}}_{0.1}=5,{\bar{X}}_{1.1}=8,n_{02}=33,n_{12}=33,n_{22}=34)\\ & \;\;+1\times P(H_{01}\;\text{is rejected},H_{02}\;\text{is not rejected}\;|\;{\bar{X}}_{0.1}=5,{\bar{X}}_{1.1}=8,n_{02}=33,n_{12}=33,n_{22}=34)\\ & \;\;+1\times P(H_{01}\;\text{is not rejected},H_{02}\;\text{is rejected}\;|\;{\bar{X}}_{0.1}=5,{\bar{X}}_{1.1}=8,n_{02}=33,n_{12}=33,n_{22}=34)\\ & \;\;+2\times P(H_{01}\;\text{and}\;H_{02}\;\text{are rejected}\;|\;{\bar{X}}_{0.1}=5,{\bar{X}}_{1.1}=8,n_{02}=33,n_{12}=33,n_{22}=34)\\ & \;=P\left({\bar{X}}_{1.2}-{\bar{X}}_{0.2}>Z(1,2),{\bar{X}}_{2.2}-{\bar{X}}_{0.2}\leq Z(2,2)\;|\;{\bar{X}}_{0.1}=5,{\bar{X}}_{1.1}=8,n_{02}=33,n_{12}=33,n_{22}=34\right)\\ & \;\;+P\left({\bar{X}}_{1.2}-{\bar{X}}_{0.2}\leq Z(1,2),{\bar{X}}_{2.2}-{\bar{X}}_{0.2}>Z(2,2)\;|\;{\bar{X}}_{0.1}=5,{\bar{X}}_{1.1}=8,n_{02}=33,n_{12}=33,n_{22}=34\right)\\ & \;\;+2\;P\left({\bar{X}}_{1.2}-{\bar{X}}_{0.2}>Z(1,2),{\bar{X}}_{2.2}-{\bar{X}}_{0.2}>Z(2,2)\;|\;{\bar{X}}_{0.1}=5,{\bar{X}}_{1.1}=8,n_{02}=33,n_{12}=33,n_{22}=34\right).\\ & \;E\left(U_{D_{a}}^{o}\;|\;{\bar{X}}_{0.1}=5,{\bar{X}}_{1.1}=8,n_{k2}=N_{2}/3\right)\\ & \;=0\times P(H_{01}\;\text{and}\;H_{02}\;\text{are not rejected}\;|\;{\bar{X}}_{0.1}=5,{\bar{X}}_{1.1}=8,n_{02}=33,n_{12}=33,n_{22}=34)\\ & \;\;+1\times P(H_{01}\;\text{is rejected},H_{02}\;\text{is not rejected}\;|\;{\bar{X}}_{0.1}=5,{\bar{X}}_{1.1}=8,n_{02}=33,n_{12}=33,n_{22}=34)\\ & \;\;+1\times P(H_{01}\;\text{is not rejected},H_{02}\;\text{is rejected}\;|\;{\bar{X}}_{0.1}=5,{\bar{X}}_{1.1}=8,n_{02}=33,n_{12}=33,n_{22}=34)\\ & \;\;+1\times P(H_{01}\;\text{and}\;H_{02}\;\text{are rejected}\;|\;{\bar{X}}_{0.1}=5,{\bar{X}}_{1.1}=8,n_{02}=33,n_{12}=33,n_{22}=34)\\ & \;=P\left({\bar{X}}_{1.2}-{\bar{X}}_{0.2}>Z(1,2),{\bar{X}}_{2.2}-{\bar{X}}_{0.2}\leq Z(2,2)\;|\;{\bar{X}}_{0.1}=5,{\bar{X}}_{1.1}=8,n_{02}=33,n_{12}=33,n_{22}=34\right)\\ & \;\;+P\left({\bar{X}}_{1.2}-{\bar{X}}_{0.2}\leq Z(1,2),{\bar{X}}_{2.2}-{\bar{X}}_{0.2}>Z(2,2)\;|\;{\bar{X}}_{0.1}=5,{\bar{X}}_{1.1}=8,n_{02}=33,n_{12}=33,n_{22}=34\right)\\ & \;\;+P\left({\bar{X}}_{1.2}-{\bar{X}}_{0.2}>Z(1,2),{\bar{X}}_{2.2}-{\bar{X}}_{0.2}>Z(2,2)\;|\;{\bar{X}}_{0.1}=5,{\bar{X}}_{1.1}=8,n_{02}=33,n_{12}=33,n_{22}=34\right). \end{array}$$

## APPENDIX B

### B.1 ILLUSTRATIONS: ADDING AN ARM WITH EXTRA PATIENTS

The figures in this section illustrate the scenario that extra patients are available when a new treatment arm is added to an existing trial. Figure B1 and Figure B2 correspond to the same setting as Figure 2 in Section 4.1 and Figure 3 in Section 4.2, except that resources are different (see Table B1).

**Table B1:** Number of patients in the illustrations. N_1_ and N_2_ are the sample size of stage one and stage two, n_k2_ is the sample size of treatment k at stage two. Equal allocation is considered in all illustrations

	Initial Number of Arms	Decision	*N*_1_	*N*_2_	*n*_*k*2_
Figure 2	2	Do not add an arm	400	300	150
		Add an arm	400	300	100
Figure B1	2	Do not add an arm	400	300	150
		Add an arm	400	300 +150	150
Figure 3	3	Do not add an arm	1200	1800	600
		Add an arm	1200	1800	450
Figure B2	3	Do not add an arm	1200	1800	600
		Add an arm	1200	1800 + 600	600
